# Supplementary figures and images for: RNAi of Complex I and V of the electron transport chain in glutamate neurons extends life span, increases sleep, and decreases locomotor activity in Drosophila melanogaster
Source: PLoS One. 2023 Jun 15;18(6):e0286828. doi: 10.1371/journal.pone.0286828 (PMC10270625; doi:10.1371/journal.pone.0286828)

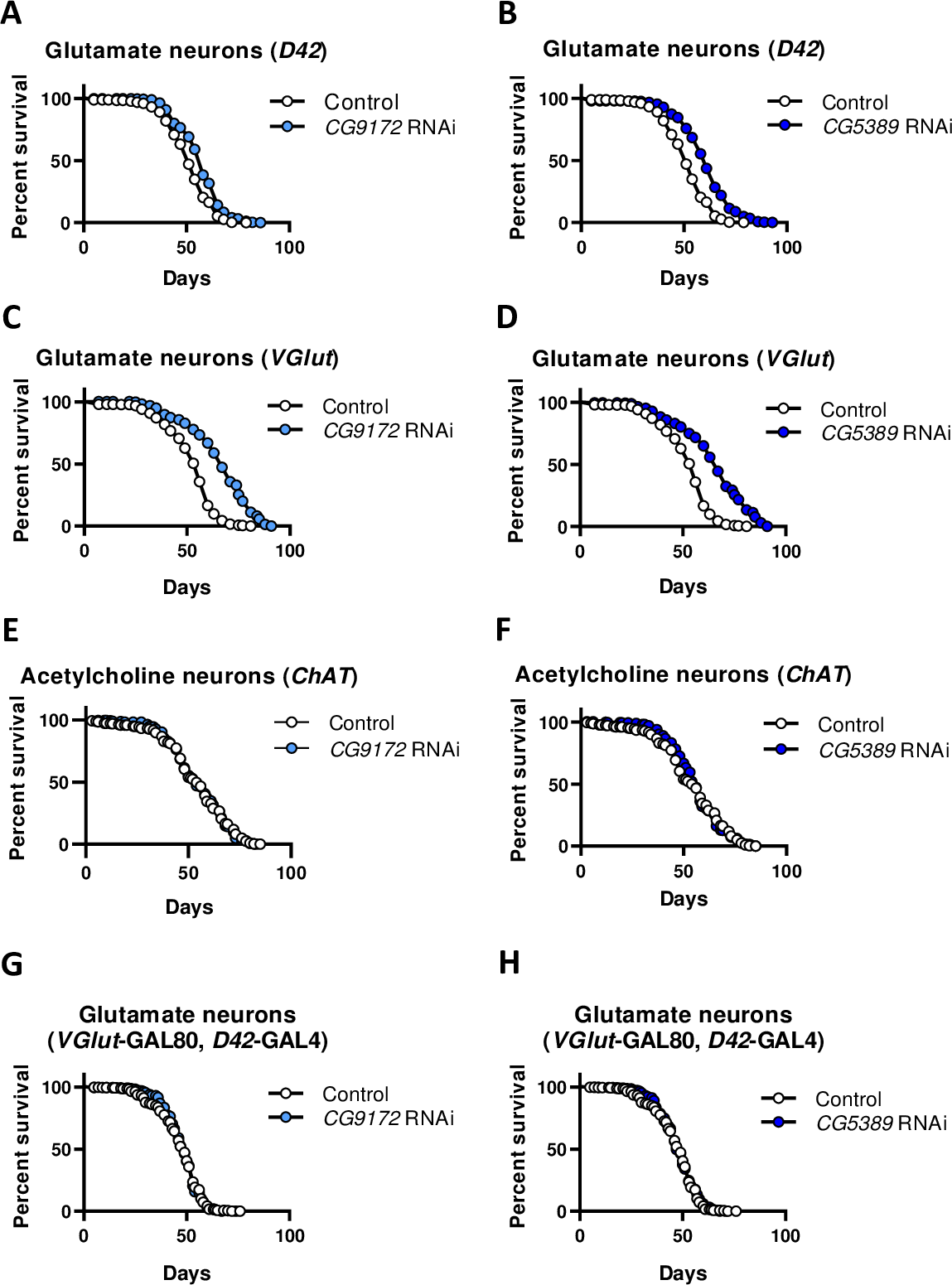

Supplement: S1 Fig — Males of the indicated GAL4 driver were mated to a white1118 control (open circles) or UAS-CG9172-RNAi (light blue), or UAS-CG5389-RNAi (dark blue) lines. (A, B) Survival curves of RNAi against CG9172 and CG5389 RNAi in D42-GAL4 glutamate neurons show comparable 11% and 17% (p < 0.0001, log rank test) life span extension. (C, D) In VGlut-GAL4 glutamate neurons, RNAi of CG9172 and CG5389 leads to an extension of 28% and 26%, respectively (p < 0.0001). (E, F) RNAi of CG9172 and CG5389 in ChAT-GAL4 acetylcholine neurons extends life span by 2% and 5%, respectively (p = 0.81, p = 54). (G, H) Activating RNAi against CG9172 and CG5389 to the set of non-overlapping set of glutamate neurons (D42-GAL4; VGlut-GAL80) minimizes life span extension to 2%, respectively (p = 0.91, p = 0.44). (TIF) [file pone.0286828.s001.tif]

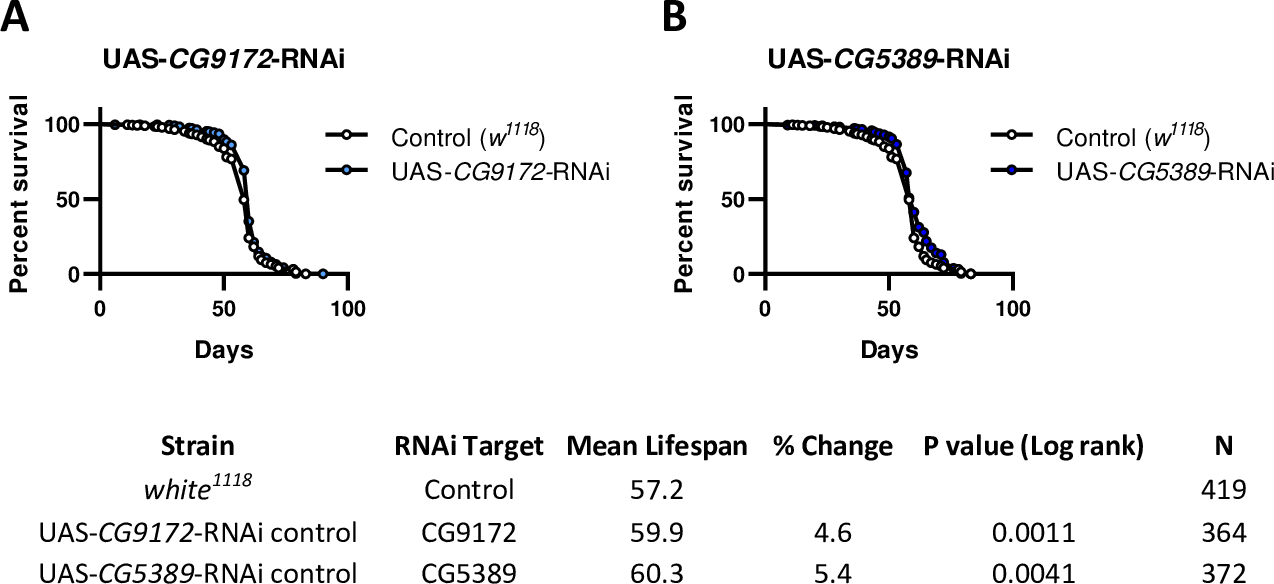

Supplement: S2 Fig — Females of the indicated UAS RNAi lines were mated to a white1118 control (closed circles) and the white1118 control line was used for comparison (open circles). (A) Survival curve of outcrossed UAS-CG9172-RNAi compared to the white1118 control shows 6% (p < 0.0001, log rank test) life span extension. (B) Survival curve of outcrossed UAS-CG5389-RNAi compared to the white1118 control shows 7% (p < 0.0001, log rank test) life span extension. (TIF) [file pone.0286828.s002.tif]

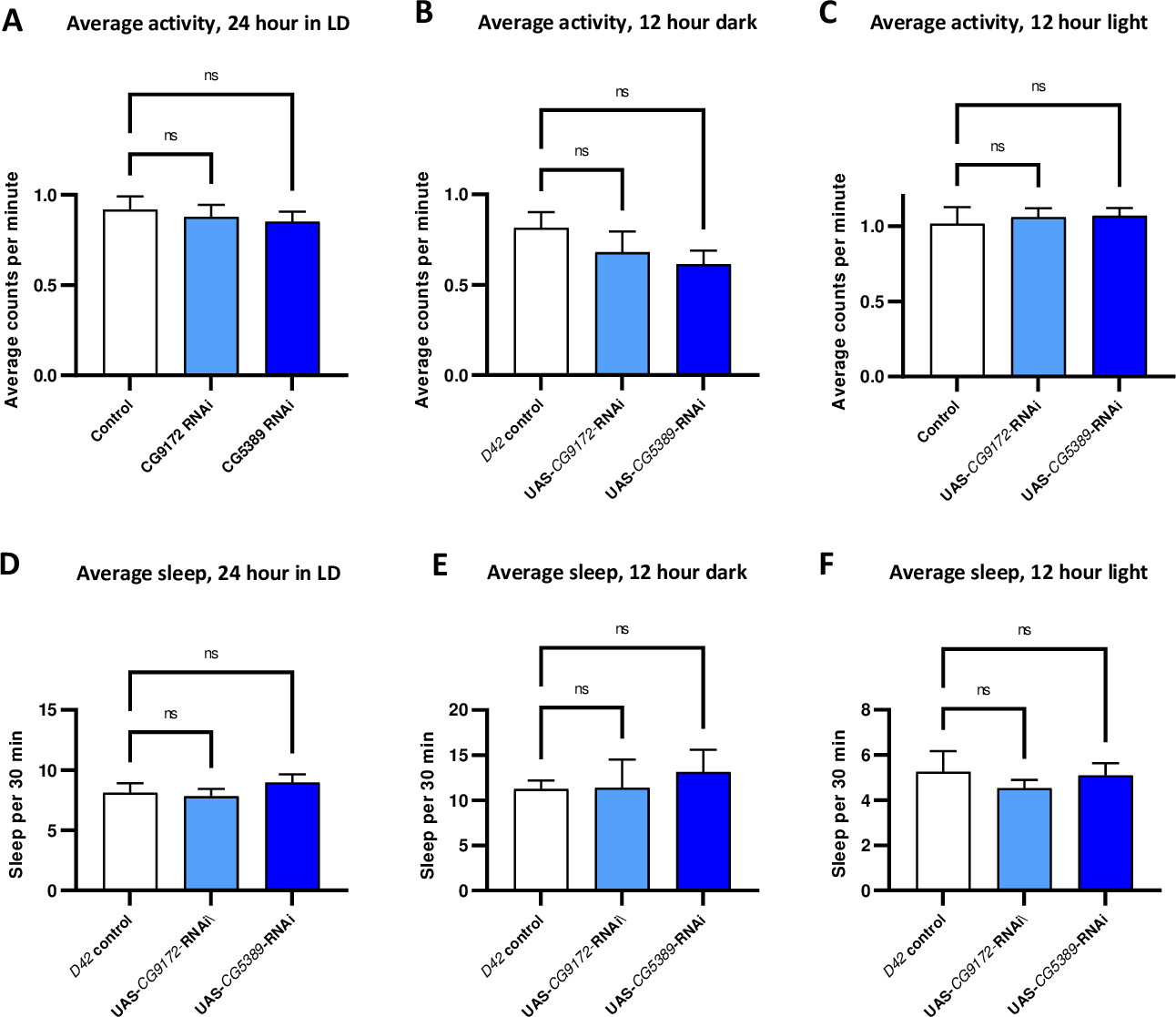

Supplement: S3 Fig — Adult male flies of either the D42-GAL4 control line (white) and unactivated RNAi to CG9172 (light blue) or CG5389 (dark blue) lines were tested for their activity and sleep. Data are presented as means +/- SEM and compared using one-way ANOVA with a Tukey’s post-hoc test. (A-C) No significant differences were observed in average activity during a 24 hour period (p = 0.89, p = 0.75), the dark phase (p = 0.57, p = 0.29), or the light phase (p = 0.92, p = 0.87) to unactivated CG9172 and CG5389 flies, respectively, in a 24 hour LD cycle. (D-F) No significant differences were observed in total sleep during a 24 hour period (p = 0.96, p = 0.68), the dark phase (p = 0.99, p = 0.17), or the light phase (p = 0.71, p = 0.99) to unactivated CG9172 and CG5389 flies, respectively, in a 24 hour LD cycle. (TIF) [file pone.0286828.s003.tif]

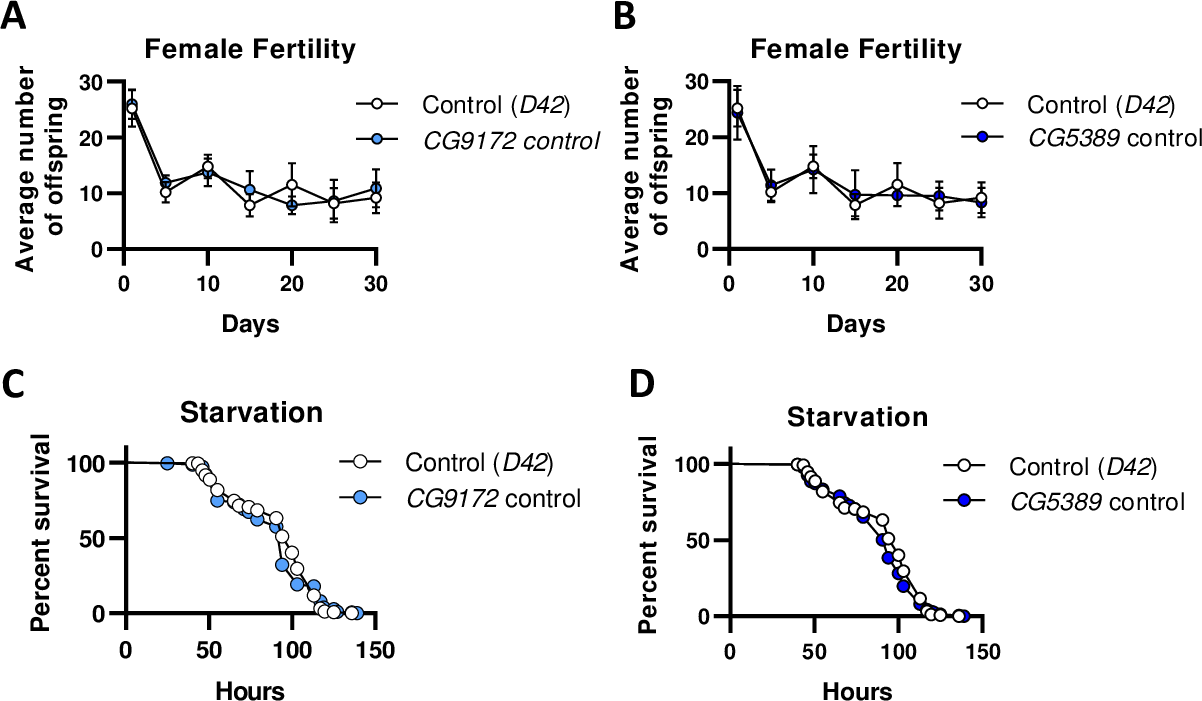

Supplement: S4 Fig — 10 day old females with the D42-GAL4 driver (open circles) were compared to females with unactivated RNAi to CG9172 (light blue) or CG5389 (dark blue) lines. (A, B) The unactivated RNAi line to CG9172 (p = 0.55, two way ANOVA) and CG5389 (p = 0.61) does not affect female fertility. (C, D). Starvation in females with unactivated RNAi to the electron transport chain genes had no effect on overall survival (CG9172, no change, p = 0.64; CG5389, p = 0.05). (TIF) [file pone.0286828.s004.tif]
